# Glucocerebrosidase deficiency promotes release of α-synuclein fibrils from cultured neurons

**DOI:** 10.1093/hmg/ddaa085

**Published:** 2020-05-11

**Authors:** Matthew E Gegg, Guglielmo Verona, Anthony H V Schapira

**Affiliations:** 1 Department of Clinical and Movement Neuroscience, UCL Queen Square Institute of Neurology, Rowland Hill Street, London NW3 2PF, UK; 2 Wolfson Drug Discovery Unit, Centre for Amyloidosis and Acute Phase Proteins, Division of Medicine, University College London, London NW3 2PF, UK

## Abstract

Mutations in the *GBA* gene, which encodes the lysosomal enzyme glucocerebrosidase (GCase), are the most important genetic risk factor for Parkinson disease (PD). GCase activity is also decreased in sporadic PD brains and with normal ageing. Loss of GCase activity impairs the autophagy lysosomal pathway resulting in increased α-synuclein (α-syn) levels. Furthermore, elevated α-syn results in decreased GCase activity. Although the role of α-syn in PD remains unclear, evidence indicates that aggregated α-syn fibrils are a pathogenic species in PD, passing between neurons and inducing endogenous native α-syn to aggregate; spreading pathology through the brain. We have investigated if preformed α-syn fibrils (PFFs) impair GCase activity in mouse cortical neurons and differentiated dopaminergic cells, and whether GCase deficiency in these models increased the transfer of α-syn pathology to naïve cells. Neurons treated with PFFs induced endogenous α-syn to become insoluble and phosphorylated at Ser129 to a greater extent than monomeric α-syn-treatment. PFFs, but not monomeric α-syn, inhibited lysosomal GCase activity in these cells and induced the unfolded protein response. Neurons in which GCase was inhibited by conduritol β-epoxide did not increase the amount of insoluble monomeric α-syn or its phosphorylation status. Instead the release of α-syn fibrils from GCase deficient cells was significantly increased. Co-culture studies showed that the transfer of α-syn pathology to naïve cells was greater from GCase deficient cells. This study suggests that GCase deficiency increases the spread of α-syn pathology and likely contributes to the earlier age of onset and increased cognitive decline associated with *GBA*-PD.

## Introduction

Mutations in the *GBA* gene are numerically the most important genetic risk factor for Parkinson disease (PD) identified to date, accounting for 5–25% of all PD cases depending on population and age (1, 2). *GBA* mutations tend to result in an earlier age of onset (~5 years) and increased cognitive decline (3–5), while Lewy body pathology is similar to idiopathic PD. *GBA* encodes for the lysosomal enzyme glucocerebrosidase (GCase) and catalyses the catabolism of the sphingolipid glucosylceramide to glucose and ceramide. GCase activity has also been reported to be decreased in idiopathic PD brains and with normal ageing (6–9), relative to other lysosomal enzymes, further implicating the enzyme in PD pathogenesis.

Cell and animal models of GCase deficiency have reported impairments in the autophagy-lysosome pathway, and in particular inhibition of macroautophagy flux (10–13), which is coincident with increased levels of α-syn (10–12, 14–18). Increased levels of wild-type (WT) or mutant α-syn has also been shown to decrease WT GCase activity (6, 7, 19, 20).

There is increasing evidence that cell-to-cell transmission of α-syn aggregates contributes to the spread of Lewy body pathology in PD brains (21). Recombinant aggregated preformed α-syn fibrils (PFFs) or pathological α-syn isolated from PD brains induces Lewy body-like pathology in naïve WT animal and cell models, such as intracellular insoluble, phosphorylated α-syn aggregates, which spread along neuronal networks (22–25). Injection of PFFs into cells of the gut also result in pathologic α-syn aggregation developing in several brain regions in a temporal fashion, which was ameliorated by vagotomy, suggesting a gut–brain axis (26).

We have used the α-syn PFF model in mouse cortical neurons (MCN) and a differentiated human dopaminergic cell line to investigate the effect on WT GCase activity and whether GCase deficiency increases spread of insoluble α-syn aggregates. Treatment with PFFs, but not monomeric α-syn, inhibited lysosomal GCase activity. The release of α-syn fibrils from neurons treated with the GCase inhibitor conduritol β-epoxide (CBE) was increased, resulting in increased transfer of α-syn pathology between differentiated dopaminergic cells.

## Results

### Lysosomal GCase activity decreased following PFF treatment

**Figure 1:**
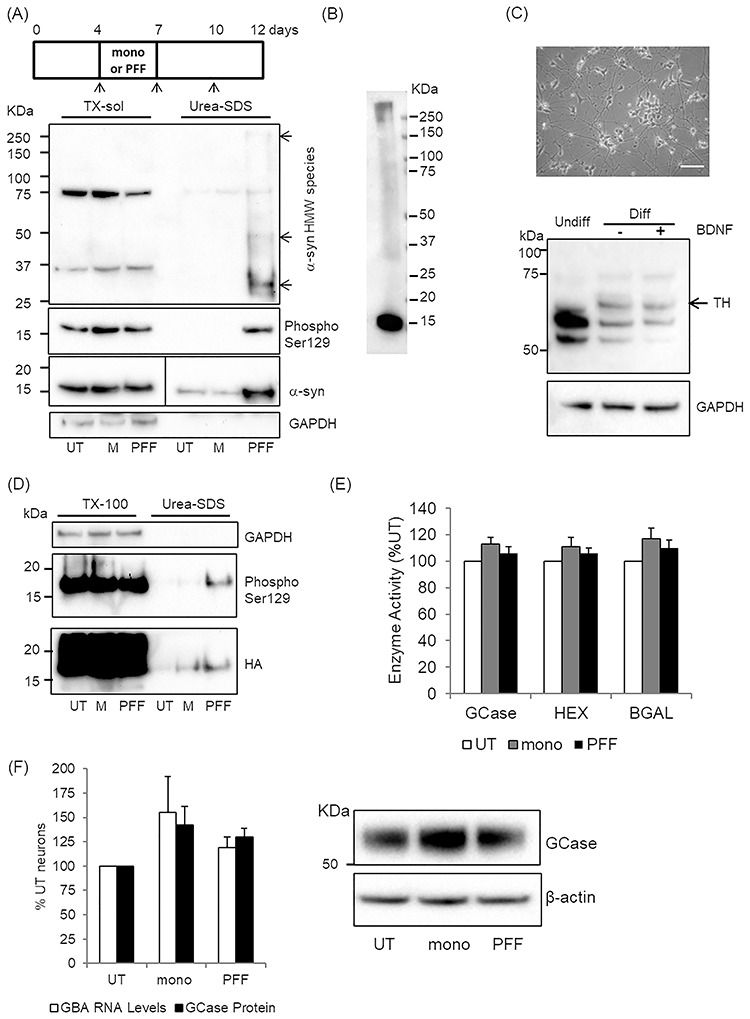
α-synuclein becomes insoluble and phosphorylated in neurons following PFF treatment. **(A)** MCN were treated with 5 μg/ml mono (M) or PFF for 3 days and media then changed (arrows) up to 12 days *in vitro*. Cells were harvested and TX-100 soluble and urea-SDS soluble fraction prepared and analysed by western blot. Fractions were probed for α-syn and α-syn phosphorylated at Ser129. For monomeric α-syn detection, the blot was cut down the ladder (solid line) and the TX-100 soluble fractions probed with 1:5000 α-syn antibody and exposed for 1.6 s; urea-SDS fraction was probed with 1:1000 α-syn antibody and exposed for 15.2 s. HMW aggregates in urea-SDS fractions are indicated by arrows. **(B)** Western blotting of recombinant PFF (10 ng) mixed with gel loading buffer. **(C)** SH-SY5Y cells were differentiated for 14 days with 30 μm retinoic acid and 10 ng/ml BDNF. Cells exhibit neuron-like morphology and no longer divide. Scale bar is 25 μm. Western blotting for TH shows induction of TH band of correct size (~60 KDa, black arrow) that is undetectable in undifferentiated SH-SY5Y cells. BDNF does not increase TH expression but does increase neurite growth and viability of differentiated SH-SY5Y cells. **(D)** Diff SH expressing HA-tagged α-syn -syn were treated with mono α-syn (M) or PFF and TX-100 soluble and urea-SDS soluble fractions prepared 8 days after initial treatment. HA-tagged α-syn detected by western blotting using an HA antibody. Treatment with M or PFF induced some α-syn to become insoluble, but only PFF treatment resulted in insoluble α-syn being phosphorylated at Ser129. GAPDH was used as a loading control. **(E)** GCase, HEX and BGAL enzyme activity in RIPA lysates was measured following mono or PFF treatment and expressed as percentage of untreated (UT) cells. Data are mean ± SEM (*n* = 7). **(F)** MCN were treated with mono α-syn or PFF and GCase mRNA levels and GCase protein levels measured 10 days after initial treatment by real-time qPCR and western blot, respectively. There is a non-significant increase in mRNA and protein levels following mono and PFF treatment. Data are expressed as percentage of untreated (UT) neurons and are mean ± SEM (GBA protein, *n* = 8; GBA mRNA, *n* = 7).

MCN were treated with 5 μg/ml monomeric α-syn (mono) or α-syn PFFs for 3 days, media changed and incubated for a further 5 days (8 days total; Fig. 1A). The vast majority of α-syn remained TX-100 soluble regardless of treatment. However as previously reported (25, 27, 28), PFF treatment resulted in increased levels of α-syn in the TX-100 insoluble fraction, which was phosphorylated at Ser129 (Fig. 1A). Phosphorylated α-syn at Ser129 was also detected by immunofluorescence in the neurites of the majority of PFF-treated cells (25, 29), and was undetectable in MCN treated with mono (Supplementary Material, Fig. S1). Note that in the insoluble fraction, neither β-actin nor glyceraldehyde 3-phosphate dehydrogenase (GAPDH) were detectable, indicating the purity of the fraction, which is important as any contamination with the soluble fraction and its much greater α-syn protein levels would limit the interpretation of results.

There was also evidence of higher molecular weight (HMW) α-syn species in the insoluble fraction following PFF treatment (arrows, Fig. 1A). Western blotting of recombinant PFF (10 ng) mixed with gel loading buffer detected two bands: one at the expected 15 kDa marker for monomeric α-syn, and a much weaker band > 250 kDa (Fig. 1B), with a similar migration to the uppermost HMW α-syn species detected in PFF-treated MCN (Fig. 1A).

To prove that at least a proportion of the insoluble α-syn we detected after PFF treatment originated from the recipient cell, and not just added PFF, we used a new method to differentiate human dopaminergic SH-SY5Y cells (Diff SH), which shows neuron-like morphology and induction of tyrosine hydroxylase (TH) protein (Fig. 1C). Note that the addition of brain-derived neurotrophic factor (BDNF) had no effect on TH expression, but improved neurite outgrowth (30). SH-SY5Y cells over expressing α-syn with a hemagglutinin (HA) tag (31) were differentiated for 4 days and treated with PFF as above. Western blotting with an HA antibody exhibited a small proportion of TX-100 insoluble HA-tagged α-syn in PFF-treated cells, which was phosphorylated at Ser129, similar to MCN (Fig. 1D).

Total cellular GCase, β-hexosaminidase (HEX) and β-galactosidase (BGAL) activities and GCase mRNA and protein levels were not significantly changed in cells treated with mono or PFF, but tended to be increased (Fig. 1E and F). Transcription factor EB (TFEB) is a master regulator of lysosomal biogenesis, including GCase and a feed forward mechanism for itself (32). TFEB mRNA levels were significantly increased in mono and PFF-treated cells [mono, 72 ± 5%, *P* < 0.01 versus control; PFF, 36 ± 8%, *P* < 0.01 versus control; *n* = 4]. Increased TFEB protein levels were also detected in the nucleus by western blot in mono and PFF-treated cells but not significantly (Supplementary Material, Fig. S2).

**Figure 2:**
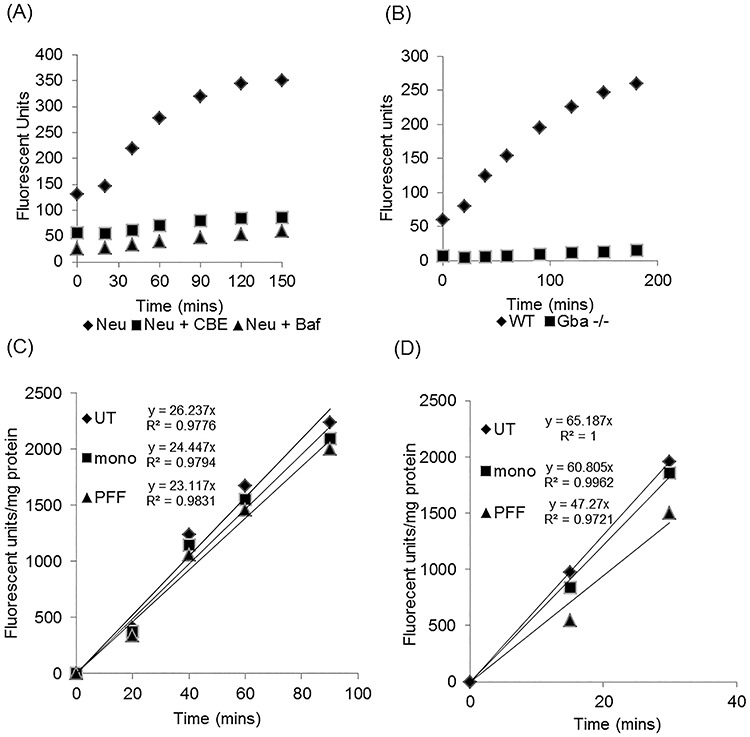
Lysosomal GCase activity is decreased following PFF treatment. **(A)** Real-time lysosomal GCase activity was measured in neurons (Neu) by following the generation of fluorescent product over time. This was abolished in cells treated with the GCase inhibitor CBE or following alkalization of lysosomes with bafilomycin A1 (Baf). Data are the mean fluorescence from three technical repeats. **(B)** Formation of fluorescent product in the *in vitro* lysosomal GCase assay was completely abolished in *Gba* -/- MEFs showing that there is no residual catalysis of the substrate by Gba2. Data are the mean fluorescence from three technical repeats. **(C)** Lysosomal GCase initial enzyme rates in MCN treated with mono or PFF. Rate equation and straightness of the line (R^2^) are indicated. Data are the CBE-sensitive rate and the mean of three technical repeats normalized to the protein content of the respective wells. **(D)** Lysosomal GCase initial enzyme rates in Diff SH treated with mono or PFF. Rate equation and straightness of the line (R^2^) are indicated. Data are the CBE-sensitive rate and the mean of three technical repeats normalized to the protein content of the respective wells.

Lysosomal GCase activity but not endoplasmic reticulum (ER) and Golgi-resident GCase can be measured in live cells using a substrate that is only taken up in to acidic vesicles, and fluoresces upon catalysis by GCase (19). Following loading of substrate there is a linear increase in fluorescent product for up to 60 min, after which enzyme activity begins to plateau (Fig. 2A). Neurites begin to retract at this point and the decrease in activity is likely a combination of prolonged incubation in Opti-minimal essential medium (Opti-MEM) and depletion of substrate. Therefore, the initial linear rate of enzyme activity was measured. Lysosomal GCase activity was abolished in MCN pre-treated with the GCase inhibitor CBE, which we have previously shown inhibits GCase activity by > 95% and impairs macroautophagy flux in MCN (12), or bafilomycin A1, which alkalizes the lysosome (Fig. 2A). Measurement of lysosomal GCase activity in *Gba1* knock-out mouse embryonic fibroblasts (MEFs) was completely abolished, indicating that formation of product is not because of cytosolic GBA2 (Fig. 2B). Lysosomal GCase activity was inhibited in MCN treated with PFF, in contrast to mono-treated or control (Fig. 2C), with the initial rate equation of lysosomal GCase activity from three independent experiments decreased by a mean of 11 ± 1% [UT, *y* = 33.91*Χ*; mono, *y* = 32.37*Χ*; PFF, *y* = 30.33*Χ*, *n* = 3].

Treatment of Diff SH with mono or PFF for the same period of time also did not decrease total GCase activity, compared with UT [mono, 110 ± 5%; PFF, 106 ± 7%, *n* = 7]. However, the initial rate of lysosomal GCase activity following PFF treatment was notably decreased (Fig. 2D), with a mean 38 ± 12% decrease, when compared with UT, for three independent experiments [UT, *y* = 74.40*Χ*; mono, y = 76.30*Χ*; PFF, *y* = 54.32*Χ*, *n* = 3]. Because the initial rate of lysosomal GCase activity was higher in Diff SH relative to MCN lysosomal GCase, activity plateaued earlier leaving a shorter time (30 min) over which to measure the initial rate. The increased activity of GCase in Diff SH versus MCN was also reflected in total cellular GCase activity (Diff SH, 470.9 ± 29.5 nmol/min/mg, *n* = 6; MCN, 245.8 ± 25.6 nmol/min/mg, *n* = 7).

### PFF treatment increased GRP78/BiP protein levels

**Figure 3:**
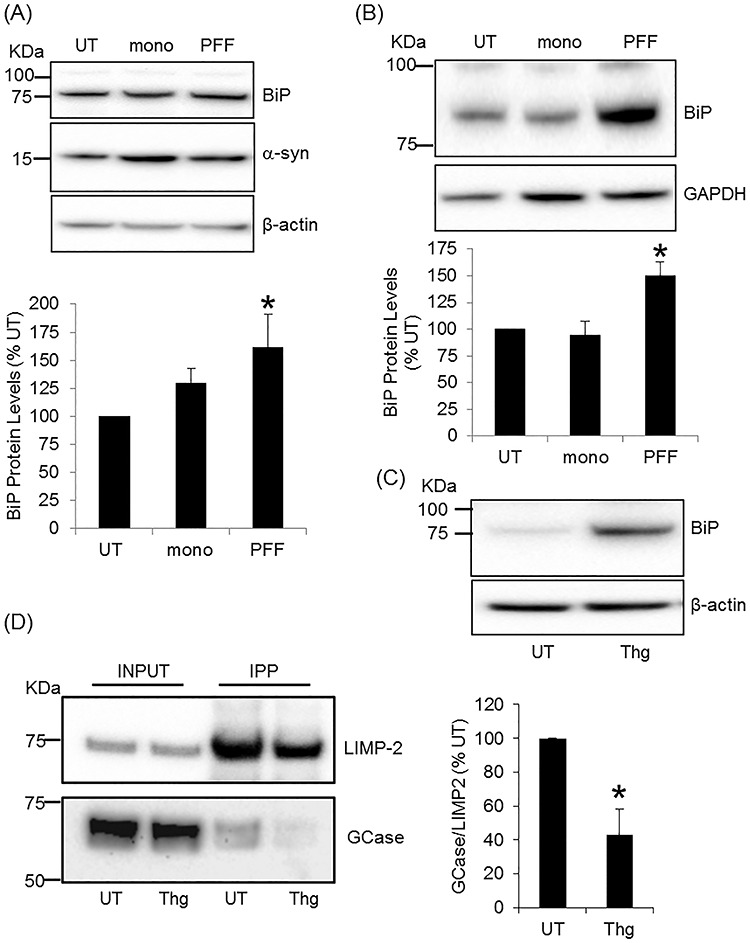
BiP levels are increased in PFF treated cells and ER stress causes GCase to dissociate from LIMP2. **(A)** Following treatment of MCN with mono or PFF, BiP and α-syn levels were measured in RIPA lysates by western blotting. BiP protein levels were normalized to β-actin and expressed as percentage untreated (UT) cells. Data are the mean ± SEM, ^*^P < 0.05 versus UT (*n* = 9). **(B)** Diff SH were treated with mono or PFF, and BiP levels were measured by western blotting. BiP protein levels were normalized to GAPDH and expressed as percentage untreated (UT) cells. Data are the mean ± SEM, ^*^P < 0.05 versus UT (*n* = 4). **(C)** SH-SY5Y cells were treated with 1 μm thapsigargin (Thg) for 1 h and induction of the unfolded protein response measured by western blotting for BiP levels. **(D)** LIMP2 was immunoprecipitated (IPP) from SH-SY5Y cells treated with Thg for 1 h. Input (2% volume of initial lysate) and immunoprecipitates were analysed by western blotting for GCase and LIMP2. GCase was expressed as a ratio to LIMP2 and the mean ± SEM calculated. ^*^P < 0.05 versus UT (*n* = 3).

The loss of lysosomal GCase activity, while total GCase activity was unchanged, might suggest that trafficking of GCase to the lysosome was impaired following PFF treatment, with accumulation of GCase in the ER and Golgi. Increased levels of WT α-syn or neurons expressing point mutations in α-syn have been shown to reduce ER to Golgi trafficking (19, 33) and induce the unfolded protein response (UPR) (34–36). The levels of soluble monomeric α-syn in MCN lysed with RIPA buffer following mono and PFF treatment for 10 days were significantly increased compared with control [mono, 232 ± 24%, *P* < 0.05; PFF, 281 ± 26%, *P* < 0.01, *n* = 5] (Fig. 3A). This was coincident with a significant increase in the UPR marker BiP in PFF-treated MCN (Fig. 3A), but not mono, when compared with control [mono, 130 ± 13%; PFF, 162 ± 29%, *P* < 0.05, *n* = 9]. BiP levels were also significantly increased in Diff SH treated with PFF but not mono (Fig 3B), when compared with control [mono, 94 ± 13%; PFF, 150 ± 13%, *P* < 0.05, *n* = 5]. Therefore, activation of BiP and the UPR following PFF treatment might account for the decrease in GCase reaching the lysosome.

As proof of principle, SH-SY5Y cells were treated with thapsigargin (Thg), a SERCA inhibitor that increases BiP levels (Fig. 3C), to assess the association of GCase with LIMP2, which transports GCase from the ER to the lysosome (37). Following Thg treatment, LIMP2 was immunoprecipitated and the amount of GCase pulled down with LIMP2 was measured by western blotting (Fig. 3D). In cells with increased BiP, the amount of GCase bound to LIMP2 was significantly decreased by 47% compared with untreated cells (Thg, 42.9 ± 15.3%, *P* < 0.05, *n* = 3). Unfortunately, neither LIMP2 nor GCase could be immunoprecipitated from MCN and we could not investigate this following PFF treatment.

### PFF treatment induced α-syn to become TX-100 insoluble but is not increased in GCase deficient cells

**Figure 4:**
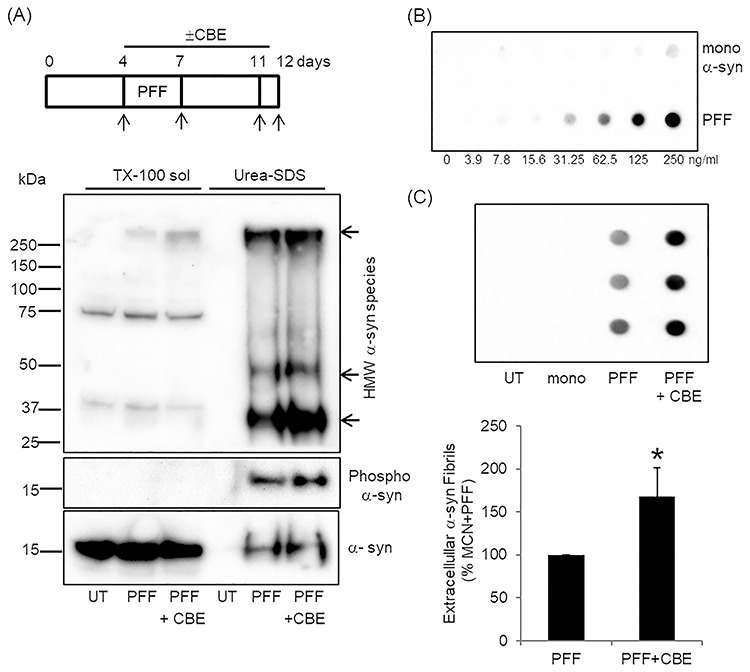
GCase deficient neurons increase the release of α-syn fibrils. **(A)** MCN were treated with PFF for 72 h and media then removed and changed (arrows) in the presence or absence of 10 μm CBE for 8 days. Culture media was conditioned for the last 24 h to assess α-syn release. TX-100 soluble and insoluble fractions (urea-SDS) were made from PFF-treated cells on day 12 and analysed by western blotting. Monomeric and HMW α-syn species (arrows) were detected in urea-SDS fractions of PFF ± CBE treated cells. Insoluble monomeric α-syn was phosphorylated at Ser129. **(B)** Example of PFF standard curve measured by dot blot. The α-syn fibril specific antibody shows a dose response for PFF and no cross-reactivity with monomeric α-syn less than 62.5 ng/ml. **(C)** Dot blot for α-syn fibrils in conditioned media from untreated (UT), mono or PFF ± 10 μm CBE-treated MCN. Conditioned media was measured in triplicate and PFF concentration calculated using a PFF standard curve (0–30 ng/ml). The mean was taken and normalized to protein content of the respective well. The bar chart shows α-syn release from PFF + CBE-treated MCN expressed as percentage PFF-treated cells from *n* = 6 independent cultures. Data are mean ± SEM. ^*^P < 0.05 versus PFF-treated MCN.

As loss of GCase activity increased α-syn levels in animal and cell models (11, 12, 14, 17, 18, 38), we measured TX-100 soluble and insoluble α-syn levels following treatment of MCN with PFF in the presence of the GCase inhibitor CBE. MCN were treated with PFF for 3 days. Media was changed and neurons incubated for a further 5 days with a final media change 24 h before neurons were harvested (Fig. 4A). The majority of α-syn remains in the TX-100 soluble fraction following PFF treatment and did not differ significantly from control (UT) or PFF + CBE neurons when quantified on lower exposure blots [PFF, 121 ± 29%; PFF + CBE, 86 ± 9%, *n* = 4]. A small proportion of α-syn became TX-100 insoluble (solubilized with SDS-urea) following PFF treatment, which was phosphorylated at Ser129 (Fig. 4A). There was no detectable contamination of the insoluble fraction by GAPDH or β-actin. Insoluble fractions were loaded as a proportion of the TX-100 soluble protein concentration, which was similar between groups [PFF, 1.08 ± 0.10 mg protein/ml; PFF + CBE, 0.93 ± 0.13 mg protein/ml; *n* = 3). When the density of the insoluble bands was measured (arbitrary units) there was no significant difference in monomeric or monomeric phospho Ser129 α-syn between PFF and PFF + CBE groups. The density of the HMW insoluble band >250 kDa was significantly higher in PFF + CBE neurons (146 ± 15%; *P* < 0.05, *n* = 3), in contrast to PFF treatment alone. There were no significant differences in the lower insoluble HMW bands (~30 and 50 kDa). The HMW band > 250 kDa observed in the TX-100 soluble fraction was very weak and observed in two out of four blots (e.g. Fig. 1A absent; Fig. 4A present).

### GCase deficient cells increase α-syn fibril release

Human midbrain neurons with WT/N370S GCase mutations and MCN treated with CBE have been shown to increase the release of monomeric α-syn into cell culture media (12, 13). The release of α-syn fibrils from the PFF-treated neurons into culture media for the last 24 h of the experiment was measured using dot blot (Fig. 4A). To prove the specificity of the antibody for α-syn fibrils, cell culture media was spiked with 0–250 ng/ml mono α-syn or PFF (Fig. 4B). The antibody detected PFF in a dose dependent manner and showed minimal cross-reactivity with mono. Analysis of conditioned media from UT or monotreated MCN showed no α-syn fibrils after 24 h. However, media from PFF-treated cells were positive for α-syn fibrils (Fig. 4C). The mean α-syn fibril concentration in conditioned media of PFF-treated MCN was 9.1 ± 3.4 ng/ml, which was 549-fold lower than the PFF concentration used to load the MCN on day 4. When the α-syn fibrils were normalized to the protein content of the well (Fig. 4C), MCN treated with PFF + CBE released significantly more fibrils compared with PFF-alone [168 ± 33%, *P* < 0.05, *n* = 6]. The release of α-syn fibrils was measured 5 days after the removal of PFF from the media, and following several media changes, greatly reducing the chance of contamination of conditioned media from when the cells were loaded with PFFs. Even if low level contamination does occur, this will be the same between PFF and PFF + CBE-treated cells. No differences in morphology or cell viability were detected among the groups [UT, 1219 ± 270 fluorescent units; mono, 1303 ± 261; PFF, 1088 ± 207; PFF + CBE, 1200 ± 205, *n* = 5] suggesting that the increased release of α-syn fibrils was not because of cell death.

**Figure 5:**
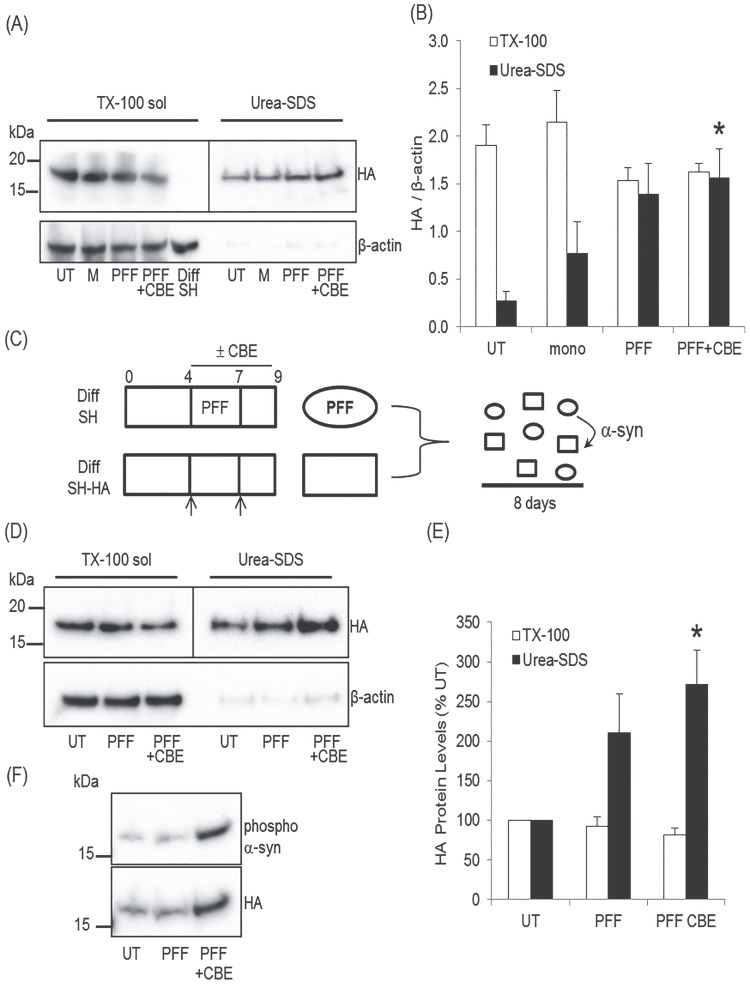
Dopaminergic cells co-cultured with GCase deficient cells pre-treated with PFFs exhibit increased insoluble α-syn. **(A)** Diff SH expressing HA-tagged α-syn were cultured with conditioned media from untreated (UT) parental Diff SH, or media taken from cells pre-treated with PFF or PFF + CBE (10 μm). Eight days after initial treatment, HA-tagged α-syn in TX-100 soluble and urea-SDS soluble fractions was measured by western blotting. β-actin was used as a loading control. A parental Diff SH lysate (lane 5) was used to show specificity of HA antibody in detecting tagged α-syn. Since the vast majority of HA-tagged α-syn was present in the TX-100 soluble fraction, membranes were cut between the soluble and insoluble fractions (line) and probed for HA separately (TX soluble, 1:20000 antibody dilution, 1 s exposure shown; urea-SDS, 1:5000, 5 s exposure shown). **(B)** HA density in soluble/insoluble fractions was expressed against β-actin and the mean ± SEM calculated for independent experiments. ^*^P < 0.05 versus UT urea-SDS (*n* = 4). **(C)** Parental Diff SH were loaded with PFF ± CBE for 72 h, and then media changed for 48 h with no PFF ± CBE. Diff SH with HA-tagged α-syn were differentiated for the same time in separate wells. Pre-treated Diff SH (oval) and HA-tagged SH (rectangle) were then passaged and mixed 1:1 and seeded into fresh culture wells for a further 8 days to allow PFF to pass from Diff SH to HA-tagged Diff SH (arrow). **(D)** Co-cultured cells were lysed and HA-tagged α-syn detected by western blot in TX-100 and urea-SDS soluble fractions. Membranes were cut between the soluble and insoluble fractions (line) and probed for HA separately (TX soluble, 1:50000 antibody dilution, 6 s exposure shown; urea-SDS, 1:10000, 38 s exposure shown). β-actin was used as a loading control. **(E)** HA density in soluble/insoluble fractions was normalized against β-actin and expressed as percentage UT of the respective fractions. Data are mean ± SEM. ^*^P < 0.05 versus UT urea-SDS (*n* = 4). **(F)** Representative blot of co-cultured urea-SDS soluble fraction probed for phospho α-syn Ser129 and HA.

### Increased insoluble α-syn induced in recipient dopaminergic cells treated with conditioned media from GCase deficient cells

Conditioned media collected from UT, PFF or PFF + CBE-treated cells was incubated with WT neurons (day 4 *in vitro*) for 72 h, media changed and incubated for a further 24 h (4-day treatment) or 7 days (10-day treatment) and TX-100 soluble and insoluble α-syn measured by western blot in recipient neurons. No insoluble α-syn was detected in any neurons treated with the three types of condition media after 4 or 10 days. Since fibril release from neurons was more that 500-fold lower than neurons initially loaded, the proportion of α-syn becoming insoluble at these time points might be below the level of detection in these experiments. The high density of cells required for detecting insoluble α-syn means that MCN do not survive longer than 15 days *in vitro*.

We therefore used Diff SH with HA-tagged α-syn to (i) increase sensitivity and (ii) as part of a co-culture model with parental Diff SH. Parental Diff SH were differentiated and treated with mono or PFF ± CBE as the MCN model (Fig. 4A). Note that the synaptic marker PSD95, as measured by western blotting, was not decreased by mono, PFF or PFF + CBE treatment [mono, 125 ± 20%; PFF, 97 ± 23%; PFF + CBE, 151 ± 33%, when compared with UT neurons (*n* = 4)]. Therefore, like MCN, this treatment regimen has no apparent effect on cell viability.

**Figure 6:**
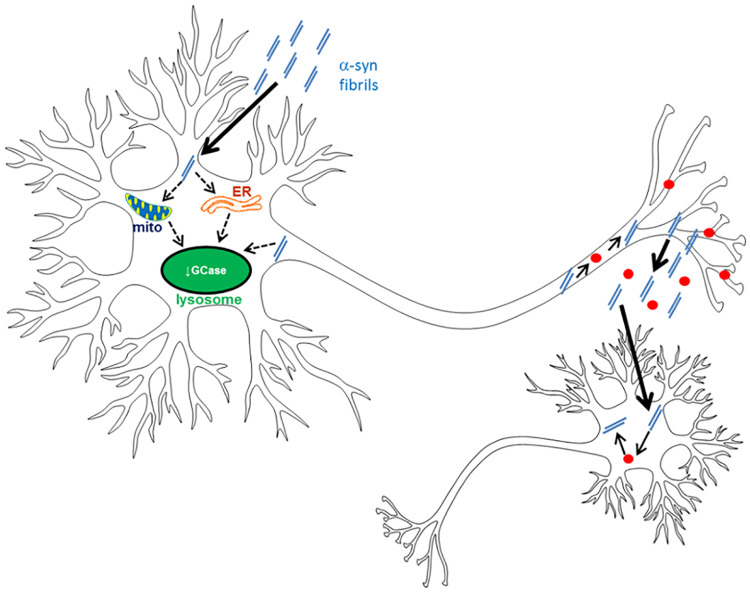
Scheme of GCase deficiency and α-syn fibrils. Treatment of neurons with α-syn fibrils (double blue lines) induces monomeric α-syn (red dots) to become insoluble and phosphorylated at Ser129. This is coincident with a reduction in lysosomal GCase activity. This loss of GCase activity could be a direct effect of fibrils on lysosomes, impairment of trafficking from the ER or oxidative stress from mitochondrial dysfunction. Neurons with GCase deficiency increase the release of α-syn fibrils from neurons, increasing the spread of pathogenic α-syn species to neighbouring cells, and might explain the earlier onset and greater cognitive decline observed in PD patients with *GBA* mutations.

The conditioned media was then incubated with Diff SH containing HA-tagged α-syn (Diff SH-HA) for 72 h, media changed and incubated for a further 7 days (10-day treatment). There was a non-significant change in TX-100 soluble HA-α-syn in cells incubated with PFF or PFF + CBE conditioned media (Fig. 5A and B), whereas TX-100 insoluble HA-α-syn was significantly increased in cells treated with PFF + CBE (Fig. 5A and B), but not mono or PFF-treated, compared with control [UT, 0.27 ± 0.10; mono, 0.77 ± 0.33; PFF, 1.39 ± 0.32; PFF + CBE, 1.56 ± 0.3, *P* < 0.05, *n* = 4]. As insoluble fractions were loaded as a proportion of protein concentration of the TX-100 soluble fraction, band density was expressed as a ratio against β-actin in the TX-100 soluble fraction. Parental Diff SH TX-100 lysate was included to prove specificity of HA antibody (Fig. 5A).

Parental Diff SH were also loaded with PFF ± CBE for 3 days, media changed and incubated for a further 2 days, before being passaged and mixed 1:1 with Diff SH-HA (same day *in vitro*, day 8) and co-cultured together in the same culture well for a further 8 days in the absence of CBE (Fig. 5C). TX-100 soluble HA-α-syn levels were not significantly changed in cells cultured with PFF + CBE differentiated SH-SY5Y (Fig. 5D and E). However, insoluble HA-α-syn was significantly increased in cells co-cultured with differentiated SH-SY5Y initially treated with PFF + CBE (Fig. 5D and E), but not PFF alone, when compared with control [PFF, 211 ± 0.49%; PFF + CBE, 272 ± 43%, *P* < 0.05, *n* = 4]. Similarly, TX-100 insoluble HA-α-syn exhibited a significant increase in phospho Ser129 when cultured with PFF + CBE cells (Fig. 5F), compared with UT [PFF, 126 ± 0.12%; PFF + CBE, 263 ± 77%, *P* < 0.05, *n* = 3].

## Discussion

We report that treatment of neurons with α-syn PFFs reduced lysosomal GCase activity. Furthermore, GCase deficient cells following PFF treatment increased the release of α-syn fibrils, which if present *in vivo*, could result in a greater spread of α-syn pathology in GBA-PD brains (Fig. 6).

Increased WT or mutant α-syn levels have been reported to decrease GCase activity and protein levels in cell and animal models and human brain (6, 7, 10, 19). We report that α-syn fibrils, but not monomeric α-syn, result in inhibition of lysosomal GCase activity in primary MCN and differentiated human dopaminergic neurons. A recent report has also shown that PFF but not monomeric α-syn inhibits GCase in hippocampal neurons over a similar time scale (39).

Total GCase protein levels (lysosomal, ER and Golgi-resident) were unchanged in our models and hippocampal primary neurons (39). This observation, coupled with our finding of real-time inhibition of lysosomal GCase activity might suggest that PFF treatment affects the trafficking of the enzyme to the lysosome. Impaired trafficking of GCase from the ER to the lysosome has been reported in human midbrain differentiated neurons with triplication of the α-syn gene (19). Activation of UPR has also been reported in these cells (34), and other neuronal models with increased α-syn (36). The decrease in lysosomal GCase following PFF treatment of MCN and Diff SH was coincident with increased BiP levels, a UPR marker, suggesting that impaired trafficking of GCase might contribute to decreased activity of the enzyme in the lysosome (Fig. 6). As proof of principal we have shown that acute induction of UPR/ER stress with Thg reduces binding of GCase to LIMP2, the protein required for transport of GCase to the lysosome. α-syn fibrils have also been reported to bind lysosomes and early, late and recycling endosomes (40), which might also affect GCase transport.

Oxidative stress from dopamine metabolism has been reported to inhibit lysosomal GCase activity (41), while PFFs have been shown to bind to mitochondria and inhibit function (25, 29), which would further enhance oxidative stress. Both effects could contribute to the apparent greater susceptibility of lysosomal GCase to PFFs in Diff SH, relative to the MCN model, and further work is required. Midbrain neurons differentiated from inducible pluripotent stem cells would appear to be an attractive model, however studies would be limited by the 20–30% yield of TH-positive cells in these cultures (11, 42), while Diff SH have the advantage of being 100% dopaminergic.

We have previously shown that inhibition of GCase by CBE in MCN and SH-SY5Y cells inhibits macroautophagy flux and impairs autophagy-lysosome reformation (12). CBE treatment has also increased α-syn levels in other cell and animal models (17, 43, 44), but not all (45–47). Timing and dosing regimens may in part explain the discrepancy. In our model, GCase was inhibited by >90% for at least 8 days. PFF treatment of these GCase inhibited MCN did not cause a further increase in insoluble or phosphorylated Ser129 monomeric α-syn or smaller HMW species (~30 and 50 kDa). However, we did observe a small but significant increase in α-syn positive insoluble higher molecular species >250 kDa in GCase deficient MCN, but was not phosphorylated at Ser129. Analysis of our recombinant PFF by western blotting showed that the >250 kDa species in both TX-100 soluble and insoluble fractions is most likely the original PFFs added to the cells, whereas the absence of the 30 and 50 kDa bands in this preparation, suggests that these species observed following PFF treatment of MCN contain endogenous α-syn. The studies on Diff SH with HA tagged α-syn do show that our PFFs can induce ‘endogenous’ α-syn to become insoluble.

Hippocampal neurons treated with CBE and PFFs also showed no further increase in insoluble α-syn species, while there was a marginal increase in phosphorylated Ser129 inclusions detected by immunofluorescence in cortical MCN and a greater increase in TH-positive midbrain neurons (39). CBE treatment of human midbrain neurons has been reported to increase TX-100 soluble HMW species, when size exclusion chromatography was employed (44). It should be noted that in this report and those described in this section (39, 44) the CBE concentration used for *in vitro* experiments inhibited GCase activity by >90%, and are therefore more likely to be modelling Gaucher disease, rather than PD with heterozygote *GBA* mutations, where 40–60% loss of GCase activity has been reported in post-mortem brains (6, 9). Titration of CBE concentrations to obtain ~50% loss of GCase activity would be useful in future. Cell models with heterozygous *GBA* mutations or RNAi with ~50% knockdown have been shown to have increased intracellular monomeric α-syn levels and/or release (11–13). Furthermore the amount of phosphorylated α-syn aggregates following PFF treatment was significantly increased in cultured neurons with Gba*^D409V/+^* mutations or ~50% knockdown of *Gba* (28, 39).

GCase deficient cells increased the release of α-syn fibrils. Inhibition of autophagy has been shown to increase the release of α-syn, in part via exosomes (48, 49). GCase deficient mouse and fly models have been shown to increase the release of exosomes/extracellular vesicles (47, 50), while lower GCase activity correlated with increased α-syn-containing exosomes in human plasma (51). Therefore, it is likely that at least some of the α-syn released in our study is present in vesicles. A limitation of this study was that we did not investigate whether the increased α-syn fibrils detected in conditioned media were present in exosomes, or whether inhibition of exosomal release by RNAi or compounds such as GW4689 can impair transfer of α-syn pathology. This will be investigated in future studies. It should also be noted that although fibril release was measured 5 days after removal of PFF from media and another media change in between, the antibody cannot distinguish between original PFFs that were added to the cells and endogenous α-syn that has formed fibrils.

The increased release of α-syn from GCase deficient cells significantly increased the amount of phosphorylated insoluble α-syn in recipient dopaminergic cells after 8 days, compared with control cells. This might suggest that if replicated in human brain, GCase deficiency can increase the spread of α-syn pathology. Since α-syn fibrils can impair WT GCase activity, it is conceivable that loss of GCase activity spreads through the brain in parallel with α-syn pathology.

However, in our model there was no significant difference between recipient cells co-cultured with PFF-treated cells alone, or PFF + CBE. An *in vivo* mouse PFF seeding model also showed that CBE inhibition of GCase did not further increase α-syn pathology after 30 days (39). Longer time periods might be required to see significant differences in α-syn pathology with GCase deficiency. The *in vivo* study did note that GCase deficiency tended to exacerbate pathology in regions with low absolute levels of α-syn pathology (39). This might have implications for the proposed gut–brain axis in PD. Overexpression of α-syn in the duodenum, or PFF treatment, has recently been shown to inhibit GCase activity, while increasing GCase expression in the duodenum slightly reduced phosphorylated α-syn and partially restored the defective gastrointestinal phenotype of these animals (52). If similar in humans, the increased spread of α-syn pathology in PD patients with *GBA* mutations might explain the earlier age of onset, the increased cognitive decline (3, 4, 53, 54) and greater cortical burden of Lewy bodies observed in PD patients with *GBA* mutations (3), in addition to *GBA* mutations increasing the risk of developing PD with dementia and dementia with Lewy bodies (55).

Increasing GCase activity in cells and animals by gene therapy or via small molecule chaperones such as ambroxol can ameliorate the increased α-syn levels observed in these models (14, 18, 56–58). Ambroxol can cross the blood brain barrier (59) and increases GCase activity by helping mutant GCase to refold in the ER and thus traffic correctly to the lysosome. Ambroxol treatment of MCN also elevates WT *GBA* mRNA levels and other transcripts encoding lysosomal proteins (60). Given these properties, it will be interesting to investigate if ambroxol treatment can improve the inhibition of lysosomal GCase by PFFs and slow down the spread of α-syn pathology in not only cell and animal PFF models with *GBA* mutations, but also WT.

In conclusion, we report that treatment of MCN or differentiated human dopaminergic SH-SY5Y cells with α-syn fibrils inhibits lysosomal GCase activity. This was coincident with increased expression of the UPR marker BiP and might reflect an impairment of GCase trafficking to the lysosome. Insoluble and phosphorylated α-syn formed after PFF treatment of neurons was not increased by inhibiting GCase with CBE. However, the release of pathogenic α-syn fibrils from neurones with GCase deficiency was significantly increased and might contribute to increased spread of α-syn pathology through the brain.

## Materials and Methods

### Mouse cortical neurons

MCN were isolated from WT C57BL6 mice (embryonic day 15) as previously described (12). This was carried out in accordance with the United Kingdom Animals (Scientific Procedures) Act of 1986. Neurons were cultured in Neuralbasal media supplemented with B-27, glutaMAX and antibiotic/mycotic solution (all Thermo Fisher) on polyornithine coated plates for up to 15 days.

### Differentiation of SH-SY5Y cells

Proliferating SH-SY5Y cells were cultured as previously described (12). SH-SY5Y cells over expressing haemagglutinin (HA)-tagged human α-syn were generated with pcDNA3.1 plasmid and selected with G418 (31). SH-SY5Y were passaged and resuspended in Neuralbasal media supplemented with B-27, glutaMAX and antibiotic/mycotic solution. Cells were seeded in this media supplemented with 30 μm retinoic acid and 10 ng/ml BDNF (R&D Systems) in to polyornithine, fibronectin (2 μg/ml) and laminin (1 μg/ml) coated plates at 3 × 10^5^ cells/ml. Media was changed every 48/72 h for up to 16 days in culture.

### Treatment of cells with α-syn

Cells were treated with 5 ug/ml mono α-syn or α-syn PFFs on day 3 or 4 *in vitro*. PFFs were sonicated for 5 s prior to dilution in culture media. Medium containing mono/PFF was incubated with cells for 72 h. Media was then removed and fresh media without α-syn added and changed as necessary. CBE treatment (10 μm) of cells started at same time as PFF treatment and was also present in subsequent media changes.

For co-culture studies, parental Diff SH were treated with PFF for 72 h, and media changed and incubated for a further 48 h (in the absence or presence of 10 μm CBE. Parental Diff SH were then lifted from the plate with accutase (ThermoFisher) and mixed 1:1 with Diff SH expressing HA-tagged α-syn (day 8 *in vitro*, same age as Diff SH). Cells were allowed to settle on freshly coated 6-well culture plates and cultured together for a further 8 days (media changes very 48/72 h) with no additional PFF or CBE treatments.

### Synthesis of PFFs

Recombinant WT α-synuclein was initially expressed in bacteria and purified with ion exchange chromatography, dissolved in sterile phosphate-buffered saline (PBS) (pH 7.4) and filter sterilized (0.22 μm) before the concentration was carefully adjusted to 2 mg/ml (Abs 0.1% = 0.412). The protein was then left for 1 week at 37°C under agitation at 250 rpm. Fibrils were isolated by centrifugation at 10 600*g* for 15 min. Fibrillar aggregates were quantified by assessment of the monomer left in the supernatant after the incubation period. The pellet was then resuspended at a concentration of 1 mg/ml in sterile PBS. A small aliquot was used to confirm the presence of genuine amyloid fibrils by Congo red staining. The same source of PFFs has successfully shown formation of α-syn pathology in mice 90 days after injection in to the dorsal striatum, with no toxicity apparent attributable to presence of endotoxin (61).

### Real-time GCase assay

Cells were treated with mono or PFF as carried out previously. For the last 24 h some wells were treated with 10 μm CBE to inhibit GCase. On day 12 *in vitro*, cells were washed with PBS and loaded with 400 μg/ml PFB-FDGluc (5-Pentafluorobenzoylamino) Fluorescein Di-β-D-Glucopyransoside (ThermoFisher) for 30 min at 37°C. Cell were washed three times and incubated in Opti-MEM and fluorescence (excitation, 488 nm; emission 520 nm) measured over time at 37°C with a fluorescent plate reader. After the experiment, media was aspirated and cells lysed with 1% (v/v) TX-100 in PBS to measure protein content of the cell with the bicinchoninic acid (BCA) protein assay (Pierce). CBE-sensitive initial rate was calculated and normalized to protein in the well. Cells were measured in triplicate.

### Total cellular lysosomal enzyme assays

Following treatment, cells were lysed in RIPA buffer (50 mm Tris, pH 8, 150 mm NaCl, 1% (v/v) NP-40, 0.5% (w/v) sodium deoxycholate, 0.1% (w/v) SDS) and GCase, HEX and β-gal measured in McIlvaine buffer with 4–methylumbelliferyl linked substrates (e.g. 4–methylumbelliferyl–β–D–glucopyranoside for GCase) as previously described (6).

### TX-100 soluble/insoluble extraction of α-syn

Following PFF treatment cells were harvested with trypsin and washed in PBS. Cell pellets were lysed in 1% (v/v) TX-100, 50 mm Tris, pH 7.5, 750 mm NaCl, 5 mm EDTA, 4 units RQ1 DNase (Promega), protease and phosphatase inhibitors (ThermoFisher) on ice for 20 min. Lysates were pelleted at 17 000 × *g* for 20 min at 4°C. TX-100 soluble fractions were placed in fresh tubes and protein concentration measured using the BCA protein assay. Insoluble pellets were solubilized in 8 m urea, 2% (w/v) SDS, 10 mm Tris, pH 7.5, 4 units RQ1 DNase, protease and phosphatase inhibitors for 15 min at room temperature. Debris was removed by centrifugation at 17 000 × *g* for 20 min.

### Western blotting

Protein was loaded on 4–12% Bis-Tris NuPAGE gels (ThermoFisher), separated by electrophoresis and transferred to Hybond PVDF membrane (GE Healthcare). For TX-100 soluble/insoluble studies, the amount of urea-SDS fraction loaded for each sample was calculated as a proportion of the protein concentration in the TX-100 soluble fraction. Because of the purity of the insoluble fraction neither β-actin nor GAPDH were detectable in the insoluble fraction. For α-syn blots, PVDF was fixed with 4% paraformaldehyde and 0.01% (v/v) glutaraldehyde for 30 min at room temperature (62). Membranes were blocked with 5% milk/PBS/0.1% (v/v) Tween 20 and incubated with the following primary antibodies: α-syn (abcam, ab1903 for mouse; ab80627 for human), α-syn phospho S129 (abcam, ab51253), β-actin (abcam, ab6276), GAPDH, (abcam, ab8245), GCase (Merck, 2E2 for human; Sigma, G4171 for mouse), GRP78/BiP (abcam, ab21685), HA (Biolegend, HA.11), histone H3 (abcam, ab1791), LIMP2 (abcam, ab16522), TFEB (abcam, ab2636), TH (abcam, ab112). Following incubation with respective HRP-conjugated secondary antibodies, blots were incubated with Immobilon Luminata Forte enhanced chemiluminescence (Merck) and images acquired and quantified using Image Lab software (BioRad). Band densities were normalized to β-actin or GAPDH.

### α-syn fibril dot blot

Culture media was conditioned for last 24 h of experiment. Media was removed from cells and centrifuged at 1000 × *g* for 5 min to remove floating cells. Cells were harvested and lysed in 1% (v/v) TX-100 in PBS and total protein content of well calculated using the BCA assay. Media was applied to PROTRAN nitrocellulose (Perkin Elmer) in triplicate using a dot blot apparatus (Bio-Rad). A PFF standard curve was also applied with serial dilution of PFF in culture media (0–30 ng/ml). Following two PBS washes, the membrane was blocked with BlockACE (BioRad) and incubated overnight at 4°C with anti-α-syn filament antibody (abcam, ab209538). Following incubation with anti-rabbit HRP-conjugated secondary antibody, membranes were incubated with Immobilon Luminata Forte enhanced chemiluminescence (Merck) and images acquired and quantified using Image Lab software (BioRad). Dot density was converted to ng/ml using the PFF standard curve and normalized to protein content of the well. The mean of each triplicate was calculated and expressed as ng fibrils/mg protein.

### Co-immunoprecipitation of LIMP2 and GCase

SH-SY5Y cells were treated with 1 μm Thg for 1 h. Cells were harvested with trypsin and LIMP2 immunoprecipitated with above-mentioned antibody on fresh cell lysates as previously described (6). Input (2% volume of initial lysate) and immunoprecipitates were analysed by western blotting for LIMP2 and GCase as carried out previously.

### qPCR

RNA was extracted from cells using the RNeasy kit (Qiagen) and converted to cDNA with QuantiTect reverse transcription kit (Qiagen). Relative expression of GCase, TFEB and and β-actin mRNA was measured with QuantiTect SYBRgreen kit (Qiagen) using a STEP One PCR machine (Applied Biosystems). β-actin or GAPDH mRNA levels were used to normalise data. Relative expression was calculated using the ΔC_T_ method. Primers: GCase F 5’-TGC TGC TCT CAA CAT CCT TGC C-3′, R 5′-TAG GTG CGG ATG GAG AAG TCA A-3′; TFEB F 5’-CCA GAA GCG AGA GCT CAC AGA T-3′, R 5′-TGT GAT TGT CTT TCT TCT GCC G-3′: GAPDH F 5′-GGA GGT GAA GGT CGG AGT-3′, R 5′-GAA GAT GGT GAT GGG ATT TC-3′; β-actin F 5’-TCT ACA ATG AGC TGC GTG TG-3′, R 5′-GGT GAG GAT CTT CAT GAG GT-3′.

### Immunofluorescence

MCN were seeded on polyornithine coated coverslips and treated with mono or PFF on day 4 *in vitro* for 8 days. Cells were fixed with 4% paraformaldehyde and permeabilized with 0.1% (v/v) TX-100 in PBS (PBS/TX). Cells were blocked with 2% goat serum in PBS/TX at room temperature for 30 min before being incubated with 1:100 α-syn phospho S129 (abcam, ab51253) in PBS/TX overnight at 4°C. Following PBS washing, and incubation with anti-rabbit alexa-488 secondary antibody (Invitrogen) cells were mounted in citifluor containing 2 μg/ml DAPI to counterstain nuclei.

### Cell viability

Cells were treated with mono or PFF in the absence or presence of 10 μm CBE in 96-well plates. On day 12 *in vitro*, Cell Titer Blue (Promega) was added to each well for 2 h and fluorescence measured on a plate reader (excitation 530 nm, emission 590 nm). The mean fluorescence was calculated from three wells.

### Statistical analyses

All data are the mean ± SEM of at least three independent experiments. Statistical significance was calculated using the Student t test or One-way ANOVA followed by Tukey HSD post hoc test where appropriate. *P* < 0.05 was considered significant.

## Supplementary Material

Supplemental_Figures_ddaa085
