# Supplementary material for: Glucocerebrosidase deficiency promotes release of α-synuclein fibrils from cultured neurons
Source: Hum Mol Genet. 2020 May 11;29(10):1716–28. doi: 10.1093/hmg/ddaa085 (PMC7322566; doi:10.1093/hmg/ddaa085)
Supplement: Supplemental_Figures_ddaa085 [file supplemental_figures_ddaa085.pdf]

Supplementary Materials

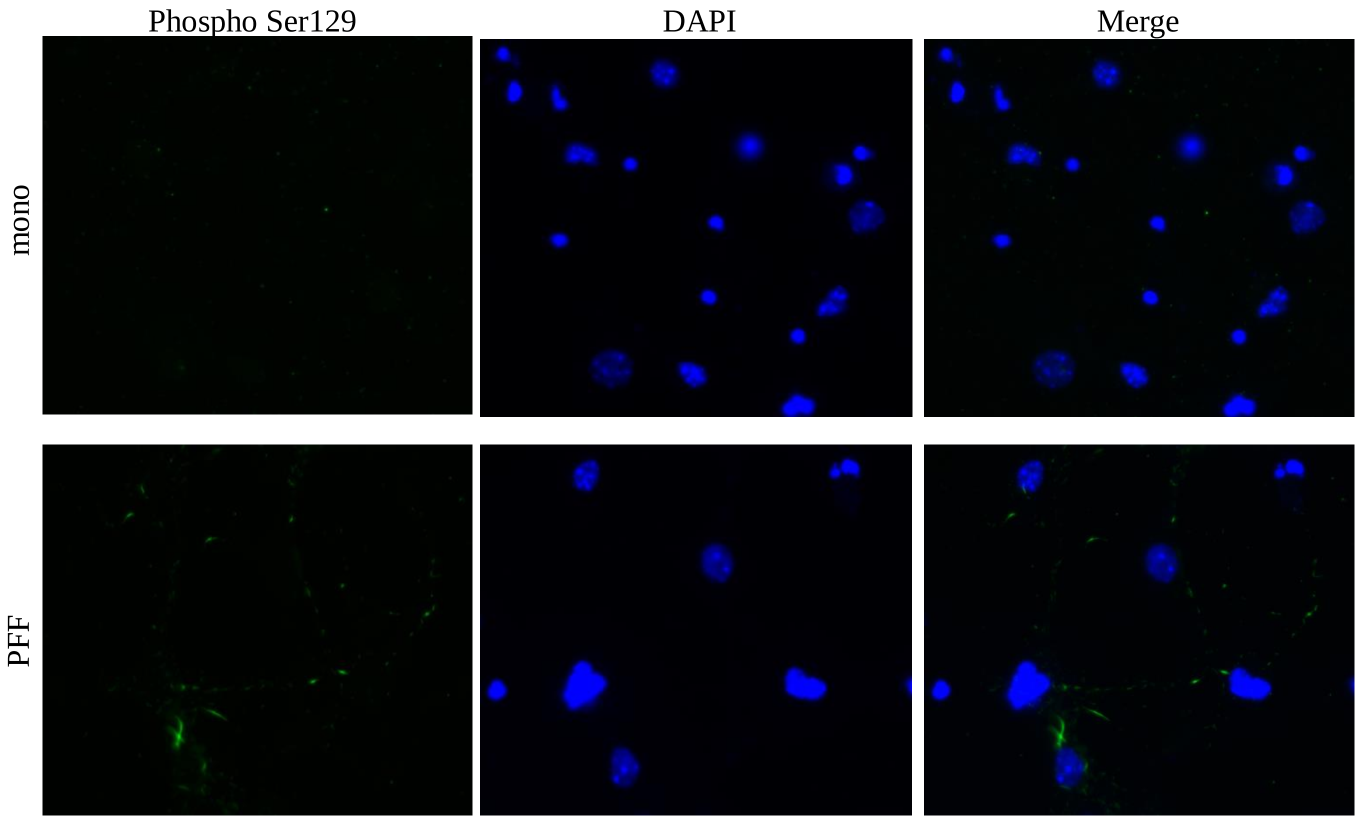

Fig SI 1. Phosphorylated α-synuclein in PFF-treated neurons. MCN were treated with mono or PFF for 8 days and phosphorylated α-syn at Ser129 (green) detected by immunofluorescence in neurites of PFF but not mono-treated neurons. Nuclei counterstained with DAPI (blue).

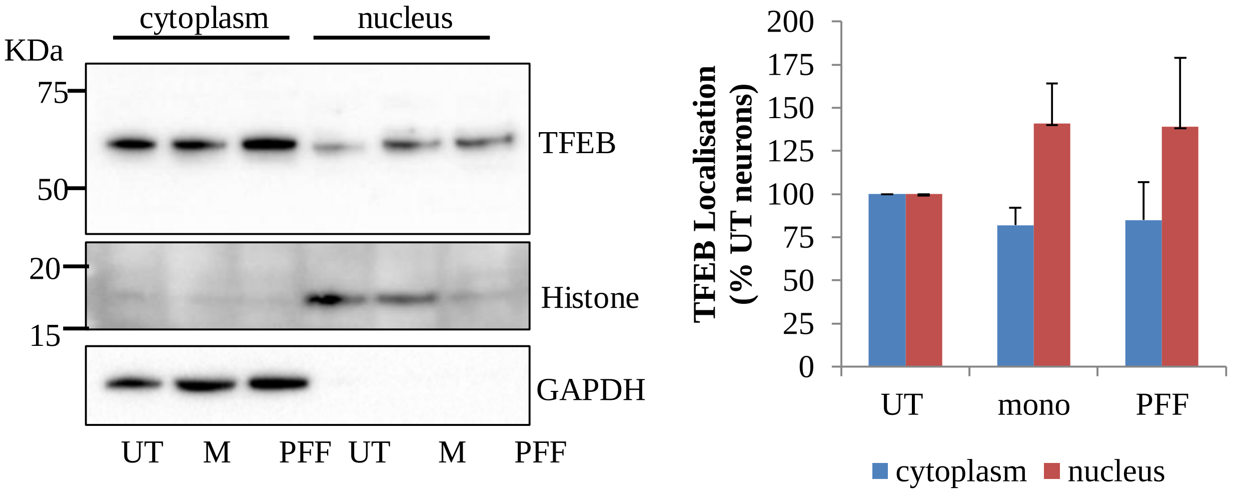

Fig SI 2. Subcellular localisation of TFEB in cortical neurons following PFF-treatment. MCN were treated with mono (M) or PFF for 8 days and cytoplasmic and nuclear fractions were prepared and analysed by western blot for TFEB. Histone and GAPDH were used for nuclear and cytoplasmic markers, respectively. TFEB density was normalised to respective markers in each fraction and data expressed as % UT neurons. Data are mean ± SEM, n = 6-8.
